# Influence of Electrostatic Forces on the Vibrational Characteristics of Resonators for Coriolis Vibratory Gyroscopes

**DOI:** 10.3390/s20010295

**Published:** 2020-01-05

**Authors:** Pengbo Xiao, Zhinan Qiu, Yao Pan, Shaoliang Li, Tianliang Qu, Zhongqi Tan, Jianping Liu, Kaiyong Yang, Wanliang Zhao, Hui Luo, Shiqiao Qin

**Affiliations:** 1 College of Advanced Interdisciplinary Studies, National University of Defense Technology, Changsha 410073, China; xiaopengbo09@nudt.edu.cn (P.X.); znqiu17@nudt.edu.cn (Z.Q.); qutianliang@nudt.edu.cn (T.Q.); zqtan@nudt.edu.cn (Z.T.); l_jianp@163.com (J.L.); yky208@nudt.edu.cn (K.Y.); luohui.luo@163.com (H.L.); 2 Shanghai Aerospace Control Technology Institute, Shanghai 201100, China; li_shaoliang@126.com (S.L.); 18916698579@163.com (W.Z.)

**Keywords:** Coriolis Vibratory Gyroscope, fused silica cylindrical resonator, frequency mismatch, Q factor, electrostatic forces

## Abstract

The Coriolis Vibratory Gyroscopes are a type of sensors that measure angular velocities through the Coriolis effect. The resonator is the critical component of the CVGs, the vibrational characteristics of which, including the resonant frequency, frequency mismatch, Q factor, and Q factor asymmetry, have a great influence on the performance of CVG. The frequency mismatch and Q factor of the resonator, in particular, directly determine the precision and drift characteristics of the gyroscope. Although the frequency mismatch and Q factor are natural properties of the resonator, they can change with external conditions, such as temperature, pressure, and external forces. In this paper, the influence of electrostatic forces on the vibrational characteristics of the fused silica cylindrical resonator is investigated. Experiments were performed on a fused silica cylindrical resonator coated with Cr/Au films. It was shown that the resonant frequency, frequency mismatch, and the decay time slightly decreased with electrostatic forces, while the decay time split increased. Lower capacitive gaps and larger applied voltages resulted in lower frequency mismatch and lower decay time. This phenomenon was theoretically analyzed, and the variation trends of results were consistent with the theoretical analysis. This study indicates that, for fused silica cylindrical resonator with electrostatic transduction, the electrostatic influence on the Q factor and frequency, although small, should be considered when designing the capacitive gap and choosing bias voltages.

## 1. Introduction

The Coriolis vibratory gyroscope (CVG) is a type of inertial device measuring angular velocity through the precession of elastic waves. The CVGs with axisymmetric shell resonators, in particular, are well known for their outstanding capabilities of high accuracy, long durability, considerable reliability, low power consumption, maintenance-free concept, and are widely used in the navigation fields and platform stabilization systems [1,2,3,4,5,6,7,8]. For example, the hemispherical resonator gyroscopes (HRGs) have claimed 30 million hours of continuous operation without a single mission failure [9].

For this type of gyroscopes, there are mainly three types of excitation and detection, including electrostatic, electromagnetic, and piezoelectric methods [10]. The representative products using electrostatic methods include the Northrop Grumman H130 series [11] and the Safran HRG Crystal^TM^ series [12,13]. The representative products with piezoelectric transduction include Watson Inc. Pro Gyro^®^ series [14] and InnaLabs Inc. GI-CVG series [15]. Wu et al. proposed a noncontact measurement system using electromagnetic excitation and microphone detection [16], which is simple as a testing apparatus, but this system can only characterize metal resonators. Due to minimal damping, electrostatic excitation and detection allow high Q factors, as in [10,17,18,19,20]. However, electrostatic excitation and detection have lower electromechanical transduction efficiency compared to piezoelectric transduction. There are several means to compensate for this, such as applying high direct current (DC) voltages [17,18,19], using gap closing mechanisms [20], relying on sub-micron transduction gaps [21,22], and adding combs [23]. On the other hand, piezoelectric transduction allows for lower motional resistance due to the higher electromechanical coupling [24,25,26,27], meanwhile requires no DC voltage application for operation, which can greatly simplify interfacing electronics. However, piezoelectric materials will inevitably introduce extra loss, which results in lower Q factors. For CVGs with axisymmetric shell resonators made from fused silica, electrostatic excitation, and detection usually outperform the rest for their low impact on the resonator, low power usage, high sensitivity, and high stability.

The axisymmetric shell resonator is the critical component of the CVG, the vibrational characteristics, including the resonant frequency, frequency mismatch, Q factor, and Q factor asymmetry, determine the overall performance of the gyroscope. The mechanical frequency mismatch and Q factor, in particular, directly determine the precision and drift characteristics of the gyroscope [28]. These vibrational parameters also affect the design of the electrical parameters, and the vibrational characteristics in practice are affected by electrical conditions in turn. Electrostatic tuning has long been recognized as an effective method for on-chip active mode matching [29,30,31,32,33,34]. To give a few examples, Darvishian et al. investigated the electrostatic frequency tuning in a birdbath shell resonator as a function of voltage, capacitive gap between the shell and electrode, electrode span angle, and height, and electrode placement and configuration using a numerical approach [34]. Ahn et al. investigated the electrostatic tuning for perfect mode-matching of a wineglass mode disk resonator gyroscope [31]. Zhang et al. investigated the mismatch compensation using electrostatic spring softening and tuning for Microscale Rate Integrating Gyroscopes (MRIGs) to operate in the whole angle mode [10]. There are also researchers investigating the effect of electrostatic forces on Q factors, but mostly for tuning fork resonators. For example, Zotov et al. electrostatically tuning the reaction force at the anchors caused by fabrication imperfection to increase the Q factor of anti-phase driven tuning fork Micro-electromechanical Systems (MEMS) [35,36]. Cheng et al. investigated the effect of polarization voltage on the measured Q factor of a multiple-beam tuning-fork gyroscope [37]. 

For CVGs with fused silica cylindrical resonators, the vibrational characteristics of the resonator will also be affected by the electrostatic forces. This paper intends to report the experimental results on the changes of resonant frequency, frequency mismatch, decay time, and decay time split under electrostatic forces, and provide theoretical analysis on these changes.

This paper comprises five sections. The theoretical analysis of the influence of electrostatic forces on the vibrational characteristics of the resonator (called vibrational characteristics in practice, VCPs) has been presented in Section 2, and comparison is made with the vibrational characteristics without electrostatic influence (called vibrational characteristics in measurements, VCMs). The methods to measure VCMs and VCPs are described in Section 3. VCMs were measured by the laser Doppler vibrometer (Polytec, Irvine, CA, USA) with acoustic excitation, while VCPs were measured with electrostatic excitation and detection. The results and discussions are presented in Section 4, and Section 5 concludes this paper with a summary of the results.

## 2. Theoretical Analysis

**Figure 1:**
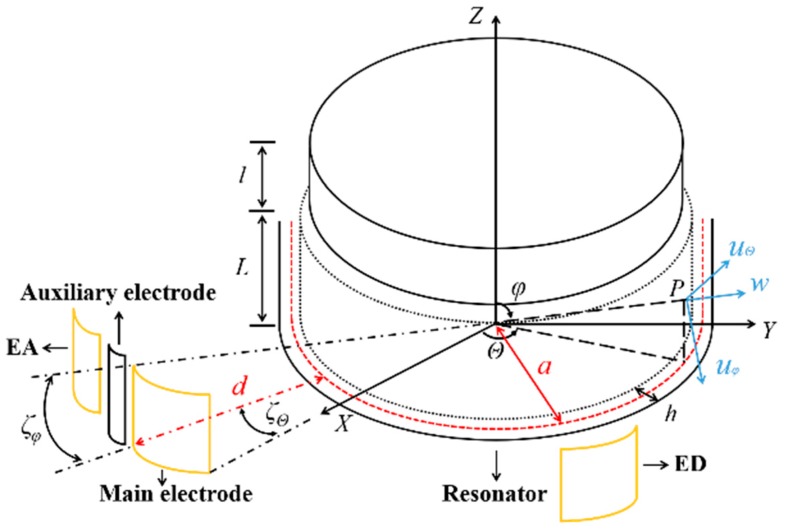
Schematic diagram of the resonator coordinate.

The kinetic energy term in the Lagrangian of a resonator was investigated using the displacement vector components in spherical polar coordinates, as shown in Figure 1. It is specified by giving the components of the displacement vector of a point *P* on the shell middle surface as a function of the spherical polar coordinates φ and Θ. The displacement vector components in spherical polar coordinates are:

(1) $$u_{\varphi }(\varphi ,\Theta ,t)=U_{\varphi }(\varphi )(w_{c}(t)\cos2\Theta +w_{s}(t)\sin2\Theta )$$

(2) $$u_{\Theta }(\varphi ,\Theta ,t)=U_{\Theta }(\varphi )(w_{c}(t)\sin2\Theta -w_{s}(t)\cos2\Theta )$$

(3) $$w(\varphi ,\Theta ,t)=W(\varphi )(w_{c}(t)\cos2\Theta +w_{s}(t)\sin2\Theta )$$

where *w_c_*(*t*) and *w_s_*(*t*) are, respectively, the radial components of the displacement vector at the equator at azimuth angles of 0° and 45°.

Considering the generic CVG equations under ideal conditions, where there is no damping, frequency mismatch or other forces and ignoring the centrifugal terms, the Lagrangian of the oscillating cylindrical shell has the form:

(4) $$L=\frac{1}{2}m_{eff}({\dot{w}}_{c}{}^{2}+{\dot{w}}_{s}{}^{2})+m_{eff}kΩ(w_{c}{\dot{w}}_{s}-{\dot{w}}_{c}w_{s})-\frac{1}{2}m_{eff}\omega^{2}(w_{c}^{2}+w_{s}^{2})$$

Among them, Ω is the system angular velocity, *k* is the angular gain, *m_eff_* is the effective mass and $m_{eff}=\frac{f(2)}{8}m$, where *f*(2) = 1.5296, *m* is the mass of the shell resonator. More detailed derivations in obtaining the values of *m_eff_* are presented in Appendix A.

When the resonator is driven and read out capacitively, additional forces should be included in the equations of motion. Considering the *k*_th_ electrode, which is placed on a spherical surface that is concentric with the shell and centered at $\varphi_{k}$ and $\Theta_{k}$, with angular widths of $\zeta_{\varphi }$ and $\zeta_{\Theta }$. If we define *E* as the electromotive force and *R* as the equivalent circuit resistance [38], we get:

(5) $$R{\dot{q}}_{k}+\frac{q_{k}}{C_{k}}=E$$

Including the Coriolis and angular acceleration terms, the centrifugal acceleration terms, the damping terms and different natural frequencies of the two modes, the equations of motion satisfied by CVGs have also been listed in Appendix A [39,40,41,42]. Therefore, Lagrange equations are

(6) $$\frac{d}{dt}\frac{\partial L}{\partial {\dot{w}}_{c}}-\frac{\partial L}{\partial w_{c}}=0$$

(7) $$\frac{d}{dt}\frac{\partial L}{\partial {\dot{w}}_{s}}-\frac{\partial L}{\partial w_{s}}=0$$

Firstly, we investigated the electrical contributions to the resonant frequency. Considering the additional electrical potential energy term in the Lagrangian, where $V_{elec}=q_{k}{}^{2}/2C_{k}$, and substituting into (6) and (7), we have:

(8) $$m_{eff}({\ddot{w}}_{c}+\frac{2}{t}{\dot{w}}_{c}+\omega^{2}w_{c})+A+\frac{q_{k}{}^{2}}{2C_{0}}\frac{\partial }{\partial w_{c}}(\frac{C_{0}}{C_{k}})=0$$

(9) $$m_{eff}({\ddot{w}}_{s}+\frac{2}{t}{\dot{w}}_{s}+\omega^{2}w_{s})+A+\frac{q_{k}{}^{2}}{2C_{0}}\frac{\partial }{\partial w_{s}}(\frac{C_{0}}{C_{k}})=0$$

*A* represents the items omitted, the explicit expressions of the values of $\frac{q_{k}{}^{2}}{2C_{0}}\frac{\partial }{\partial w_{c}}(\frac{C_{0}}{C_{k}})$, $\frac{q_{k}{}^{2}}{2C_{0}}\frac{\partial }{\partial w_{s}}(\frac{C_{0}}{C_{k}})$, and *A* are presented in Appendix B. Substituting these expressions into (8) and (9), we have:

(10) $$\begin{array}{l} m_{eff}{\ddot{w}}_{c}+m_{eff}\left[\omega^{2}-\frac{C_{0}E^{2}}{2m_{eff}d^{2}}(\gamma_{2}-\gamma_{1}{}^{2}\frac{\sin^{2}\zeta_{\Theta }}{\zeta_{\Theta }{}^{2}})\right]w_{c}\\ +m_{eff}(\frac{2}{t}+\frac{1}{RC_{0}\omega }\gamma_{1}{}^{2}\frac{\sin^{2}\zeta_{\Theta }}{\zeta_{\Theta }{}^{2}}\frac{C_{0}E^{2}}{2m_{eff}\omega d^{2}}){\dot{w}}_{c}+A=0 \end{array}$$

(11) $$\begin{array}{l} m_{eff}{\ddot{w}}_{s}+m_{eff}\left[\omega^{2}-\frac{C_{0}E^{2}}{2m_{eff}d^{2}}(\gamma_{2}-\gamma_{1}{}^{2}\frac{\sin^{2}\zeta_{\Theta }}{\zeta_{\Theta }{}^{2}})\right]w_{s}\\ +m_{eff}(\frac{2}{t}+\frac{1}{RC_{0}\omega }\gamma_{1}{}^{2}\frac{\sin^{2}\zeta_{\Theta }}{\zeta_{\Theta }{}^{2}}\frac{C_{0}E^{2}}{2m_{eff}\omega d^{2}}){\dot{w}}_{s}+A=0 \end{array}$$

Therefore,

(12) $${\omega^{\prime }}^{2}=\omega^{2}-\frac{C_{0}E^{2}}{2m_{eff}d^{2}}(\gamma_{2}-\gamma_{1}{}^{2}\frac{\sin^{2}\zeta_{\Theta }}{\zeta_{\Theta }{}^{2}})$$

(13) $$\frac{1}{t^{\prime }}=\frac{1}{t}+\frac{1}{RC_{0}\omega }\gamma_{1}{}^{2}\frac{\sin^{2}\zeta_{\Theta }}{\zeta_{\Theta }{}^{2}}\frac{C_{0}E^{2}}{4m_{eff}\omega d^{2}}$$

Similarly, we have

(14) $$\Delta \omega^{\prime }=\Delta \omega +(\gamma_{2}\frac{\sin2\zeta_{\Theta }}{2\zeta_{\Theta }}-\gamma_{1}{}^{2}\frac{\sin^{2}\zeta_{\Theta }}{\zeta_{\Theta }{}^{2}})\frac{C_{0}E^{2}}{2m_{eff}\omega d^{2}}$$

(15) $$\Delta (\frac{1}{t}{)}^{\prime }=\Delta (\frac{1}{t})+\frac{1}{RC_{0}\omega }\gamma_{1}{}^{2}\frac{\sin^{2}\zeta_{\Theta }}{\zeta_{\Theta }{}^{2}}\frac{C_{0}E^{2}}{2m_{eff}\omega d^{2}}$$

where $\omega =\sqrt{\frac{\left(\omega_{1}{}^{2}+\omega_{2}{}^{2}\right)}{2}}$, $\omega \Delta \omega =\frac{\omega_{2}{}^{2}-\omega_{1}{}^{2}}{2}$, $\frac{1}{t}=\frac{1}{2}(\frac{1}{t_{1}}+\frac{1}{t_{2}})$, and $\Delta (\frac{1}{t})=(\frac{1}{t_{2}}-\frac{1}{t_{1}})$. $\omega_{1}$, $\omega_{2}$ are, respectively, the angular frequency of the resonator excited in the low-frequency principal axis and the high-frequency principal axis, while $t_{1}$, $t_{2}$ are, respectively, the decay time constant of the resonator excited in the low-damping axis and the high-damping axis. 

In addition, the relation between angular frequency and resonant frequency is $\omega_{1}=2\pi f_{1}$, and the relation between the Q factor and decay time is $Q=\pi f_{1}t_{1}$[43]. Therefore, $\omega_{2}=2\pi f_{2}$ and we let ω=2πf, Δω=2πΔf. $f_{1}$ and $t_{1}$, in particular, are respectively the resonant frequency and the decay time constant detected in Section 4.

## 3. Experiments and Methods

**Figure 2:**
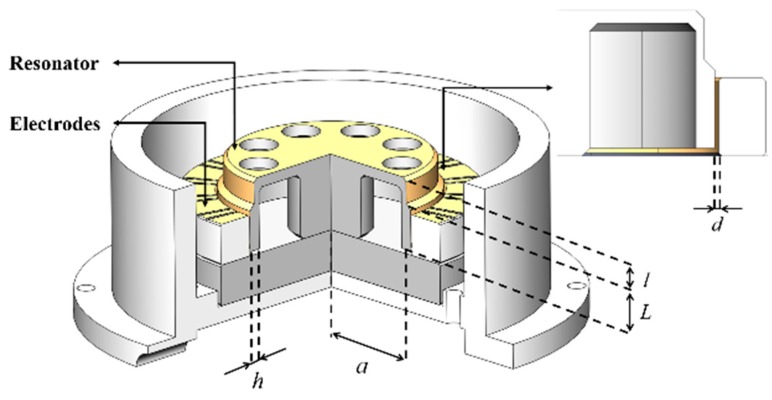
The sectional view of the resonator and the electrodes.

Our research group has reported fused silica cylindrical resonators with the Q factor approaching 10^6^ in 2016 [44] and 3 × 10^6^ in 2019 [45]. In this research, a fused silica cylindrical resonator with a high Q factor was fabricated in the same way. For electrostatic excitation and detection, the outer surface of the resonator was coated with Cr/Au (~20/60 nm) film by magnetron sputtering. The resonator was then fixed on a fused silica base through its supporting rod, and a cylindrical ring with laser-cut electrodes was attached on the base outside the resonator. The gap between the resonator and the ring was nearly 20 μm. The main electrodes were used to excite or detect resonator vibration, while the auxiliary electrodes were grounded to reduce signal interference, as shown in Figure 2. Table 1 presents some dimensions of the resonator, as well as some parameters of the electrostatic excitation and detection system, where *L* and *l* are, respectively, the height of corresponding cylinders, and *h* is the width of the resonator. The resonator was characterized in a vacuum chamber with a pressure of 0.01 Pa, and it was placed on an optical table to avoid environmental vibrations.

### 3.1. Vibrational Characteristics without Electrostatic Influence

**Figure 3:**
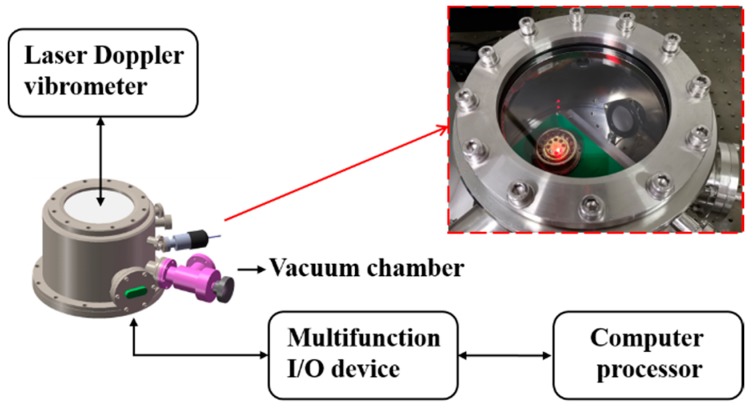
The diagram of the experimental setup used for the testing of both the VCMs and the VCPs.

For the measurement of the vibrational characteristics without electrostatic influence (VCMs), the resonator system should be isolated from the applied voltage. A laser Doppler vibrometer (Polytec, Irvine, CA, USA) was used to measure the resonant frequency, frequency mismatch, Q factor (decay time), and Q factor asymmetry (decay time split). The resonator was excited by an acoustic source and its vibration detected by the laser Doppler vibrometer. There were material anisotropy and manufacturing errors; therefore, the resonator shows a pair of principal axes of vibration (low-frequency principal axis and high-frequency principal axis), resulting in a natural frequency mismatch. The excitation direction and the low-frequency principal axis had already been aligned in the same orientation before the measurement. The diagram of the experimental setup is shown in Figure 3, and the testing procedure of the VCMs has been described in detail in [16,44,46].

### 3.2. Vibrational Characteristics with Electrostatic Influence

**Figure 4:**
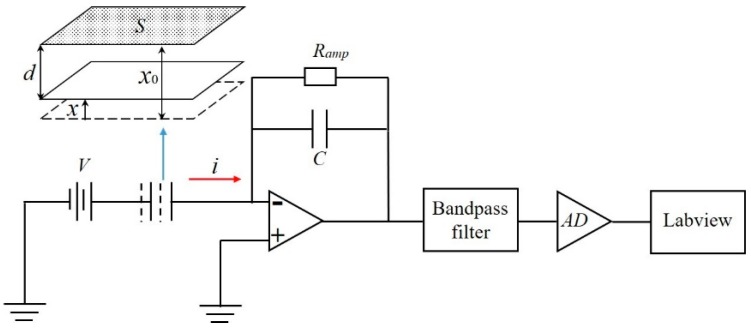
The schematic of the electrostatic detection.

For the measurement of the vibrational characteristics in practice (VCPs), the resonator system was tested under electrostatic excitation and detection. Electrostatic excitation is based on parallel plate capacitance where the two charged parallel plates produce an attractive force, and the electrostatic force can be obtained by applying an appropriate voltage on the electrodes. Electrostatic detection is also based on parallel plate capacitance where the two movable plates can charge or discharge, hence producing a measurable current for the following conditioning circuits [47]. The outside surface of the resonator and the main electrodes formed parallel plate capacitors, which were used to excite or detect the displacement of the resonator from different directions. Figure 4 shows the schematic of electrostatic detection, including the C/V converter, bandpass filter, analog to digital (AD) converter, and LabVIEW process program. The upper plate represents one of the main electrodes and the bottom plate represents the outside surface of the resonator.

The capacitance for a parallel plate capacitor is [48]:

(16) $$C=\frac{\varepsilon S}{d}$$

where ε is the permittivity of the material between two movable plates, *S* is the area of the plates, *d* is the actual gap when the resonator vibrates, *x*_0_ is the initial gap between two movable plates, and *x* is the displacement of the bottom plate. A variation in the gap between two movable plates causes a variation in the capacitance, resulting in the variation of the current. The series expansion of the current *i* around *x* = 0 is:

(17) $$i=\frac{\varepsilon S}{x_{0}{}^{2}}V\dot{x}+2\frac{\varepsilon S}{x_{0}{}^{3}}Vx\dot{x}+o(x^{2})$$

where *V* is the high DC voltage applied to the resonator. Because the magnitude of the resonant surface *x* is far less than the initial gap *x*_0_, the higher-order terms o(*x*^2^) are negligible. Substitute x=asinωt into (17); the cross term $x\dot{x}$ splits into a DC component and a 2ω frequency component, which can both be eliminated by the bandpass filter. The output current signal is

(18) $$i=\frac{\varepsilon S}{x_{0}{}^{2}}Va\omega \cos\omega t$$

The output voltage signal is then:

(19) $$U=R_{amp}\frac{\varepsilon S}{x_{0}{}^{2}}Va\omega \cos\omega t$$

where *R_amp_* is the resistance.

Using a multifunction I/O device, the actuating capacitors were connected to voltage sources. Excitation signals were generated by the multifunction I/O device with the controlled program designed and operated in the LabVIEW software, and all the relative parameters could be easily adjusted. Detection signals from sensing capacitors were collected by the multifunction I/O device and processed by the LabVIEW program, as shown in Figure 3.

The testing procedure of the VCPs was as follows. A pair of ring electrodes EA, along with the low-frequency principal axis was used for actuation, while the pair ED in quadrature with EA was used for detection. A sweeping voltage signal was applied to EA and the sweeping frequency data was recorded from ED. The resonant frequency *f* was then obtained through Fast Fourier transform. As for Q factor measurement, a sinusoidal voltage signal with the resonant frequency was applied to EA, and the signal was then cut off., and the ring-down time was recorded. The measurement for the resonant frequency and decay time was repeated for the high-frequency principal axis; hence, the frequency mismatch and the decay time split were acquired.

## 4. Results and Discussion

### 4.1. Vibrational Characteristics in Measurements

Table 2 presents all the VCMs detected by the laser Doppler vibrometer. Series 1 represents the VCMs excited in the low-frequency principal axis. Series 2 represents the VCMs excited in the high-frequency principal axis. Series Δ represents the variations between Series 1 and Series 2. 

**Figure 5:**
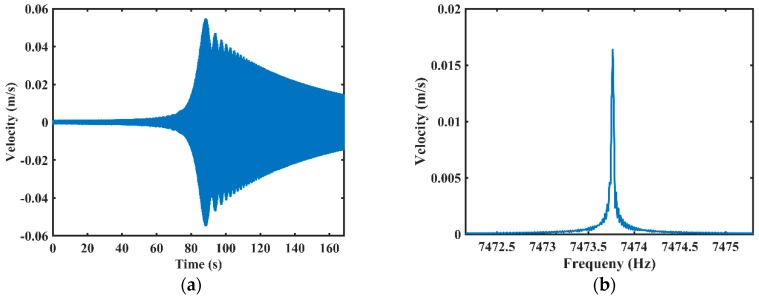
The results of the sweeping frequency detected by the laser Doppler vibrometer: (**a**) The vibration velocity detected by the laser Doppler vibrometer; (**b**) Fast Fourier transform (FFT) results of the vibration velocity.

The sweeping measurements detected by the laser Doppler vibrometer are shown in Figure 5. When applying a sweeping signal to the acoustic source, the resonator was excited and gradually reached the maximum value, about 55 mm/s, as shown in Figure 5a. Then, the vibration velocity gradually decayed as the frequency of the excitation signal deviated from the resonant frequency. The sweeping frequency data was processed by a Fast Fourier Transform (FFT) program, and the resonant frequency is about 7473.767 Hz, as shown in Figure 5b.

**Figure 6:**
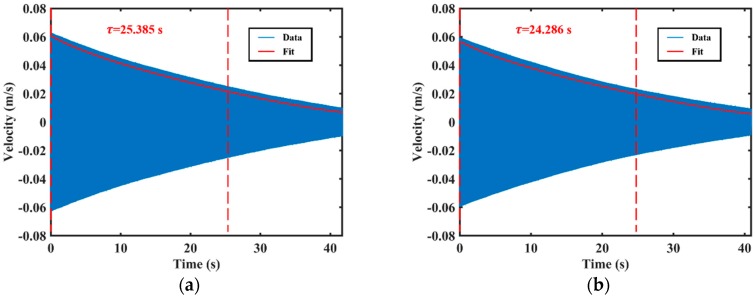
The decay time constant measured by ring-down time method: (**a**) The decay time constant excited by the low-frequency principal axis; (**b**) The decay time constant excited by the high-frequency principal axis.

The ring-down time measurement results are shown in Figure 6. The decay time constant of the resonator (low-frequency axis excited) is about 25.385 s, as shown in Figure 6a. The Q factor is approximately 5.960 × 10^5^, calculated by the equation described in [43]. Similarly, the resonant frequency of the high-frequency principal axis was measured to be about 7474.133 Hz, the decay time constant was about 24.286 s, as shown in Figure 6b. The Q factor is approximately 5.703 × 10^5^.

**Figure 7:**
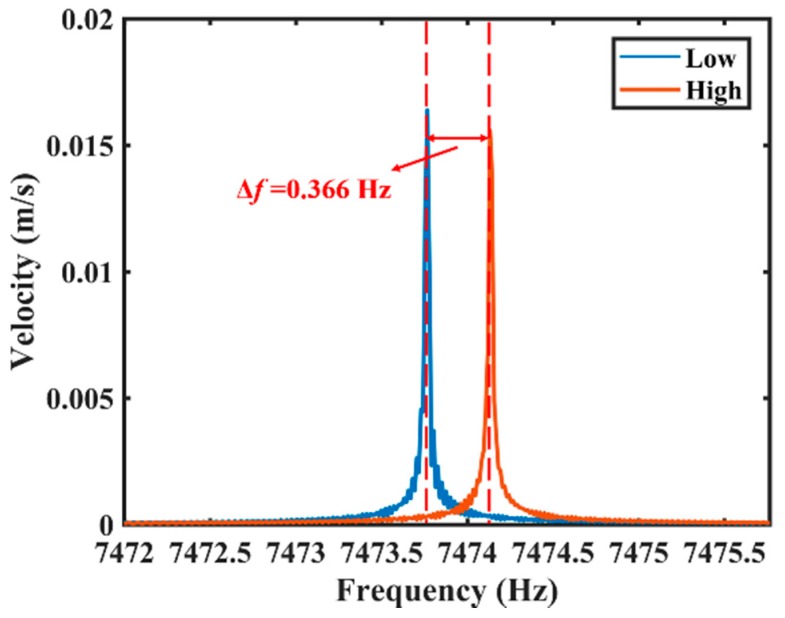
The FFT results of the vibration velocity of two vibrating modes with different frequencies. The blue line represents exciting in the direction of the low-frequency principal axis, and the red line represents exciting in the direction of the high-frequency principal axis.

The variation of the decay time is about 1.099 s, and the variation of the Q factor is about 2.578 × 10^4^. The frequency mismatch of the resonator is about 0.366 Hz, as shown in Figure 7.

### 4.2. Vibrational Characteristics in Practice

Table 3 presents all the VCPs detected by electrostatic excitation and detection. Series 1 represents the VCPs excited in the low-frequency principal axis. Series 2 represents the VCPs excited in the high-frequency principal axis. Series Δ represents the variations between Series 1 and Series 2.

**Figure 8:**
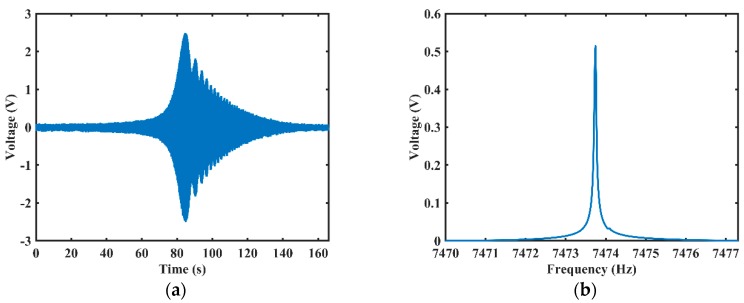
The results of frequency sweeping detected by electrostatic excitation and detection: (**a**) The output voltage signal of the ED; (**b**) Fast Fourier transform (FFT) results of the output voltage signal.

The sweeping measurements detected by electrostatic excitation and detection are shown in Figure 8. When applying a sweeping signal to EA, the output voltage signal of ED reached its maximum magnitude, about 2.5 V, as shown in Figure 8a. Then, the output voltage signal of ED gradually decayed as the frequency of the excitation signal deviated from the resonant frequency. The sweeping frequency data was processed by an FFT program, and the resonant frequency is about 7473.745 Hz, as shown in Figure 8b.

**Figure 9:**
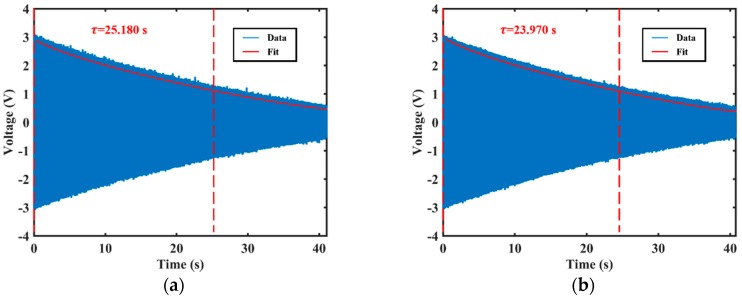
The decay time constant measured by ring-down time method: (**a**) The decay time constant excited by the low-frequency axis; (**b**) The decay time constant excited by the high-frequency axis.

The ring-down time measurement results are shown in Figure 9. The decay time constant of the resonator (low-frequency axis excited) is about 25.180 s, as shown in Figure 9a. The Q factor is approximately 5.912 × 10^5^. Similarly, the resonant frequency of the high-frequency principal axis was about 7474.085 Hz, the decay time constant was about 23.970 s, as shown in Figure 9b. The Q factor is approximately 5.628 ×10^5^.

**Figure 10:**
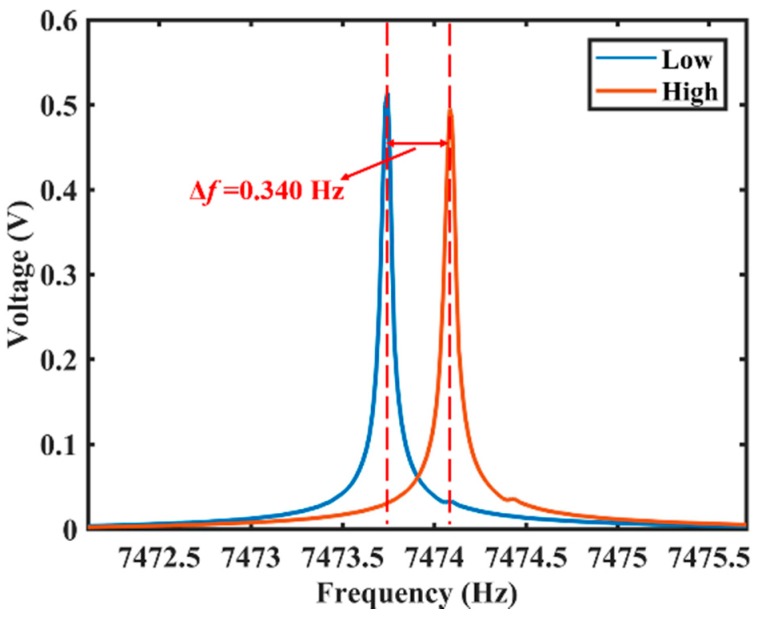
The FFT results of the output voltage signal of two vibrating modes with different frequencies. The blue line represents exciting in the direction of the low-frequency principal axis, and the red line represents exciting in the direction of the high-frequency principal axis.

The variation of the decay time is about 1.210 s, and the variation of the Q factor is about 2.838 × 10^4^. The frequency mismatch of the resonator is about 0.340 Hz, as shown in Figure 10.

### 4.3. Results and Comparisons between Analysis and Experiments

Table 4 presents the results and comparisons between the measured VCMs and the VCPs, where *f*_1_ is the resonant frequency of the low-frequency principal axis, *t*_1_ is the decay time, *f*_2_ ‒ *f*_1_ is the frequency mismatch, and *t*_2_ ‒ *t*_1_ is the decay time split. The resonant frequency in measurements and in practice of the resonator are, respectively, 7473.767 Hz and 7473.745 Hz, which decreases by 0.022 Hz. The frequency mismatch in measurements and in practice are respectively 0.366 Hz and 0.340 Hz, which decreases 0.026 Hz. The decay time in measurements and in practice are, respectively, 25.385 s and 25.180 s, which decreases by 0.205 s. The decay time split in measurements, and in practice are, respectively, 1.099 s to 1.210 s, which increases 0.111 s. Therefore, electrostatic forces do have an influence on vibrational characteristics, and the influence is relatively minor but still cannot be ignored.

Table 5 presents all the results and comparisons between the VCMs and the theoretical vibrational characteristics, and between the VCMs and the VCPs. Using the vibrational characteristics listed in Table 3, the corresponding *f*, Δf, $\frac{1}{t}$, and $\Delta (\frac{1}{t})$ in practice can be calculated, and the comparisons between the VCMs and the VCPs are listed in Variation 1. With parameters in Table 1 and the measured resonant frequency without electrostatic influence, we can calculate the $\omega^{\prime }$ according to Equation (12). Therefore, when the resonant frequencies in measurements of the resonator are respectively 7473.767 Hz and 7474.133 Hz in two axes, *f* equals to 7473.950 Hz, and the theoretical result *f* ‘ equals to 7473.927 Hz, which decreased by 0.023 Hz. According to Equation (14), when the Δf in measurements of the resonator is 0.366 Hz, the theoretical result $\Delta f^{\prime }$ equals to 0.339 Hz, which decreased by 0.027 Hz. According to Equation (13), when the decay time in measurements of the resonator are respectively 25.385 s and 24.286 s in two axes, $\frac{1}{t}$ equals to 0.0403 s^−1,^ and the theoretical result $\frac{1}{t^{\prime }}$ equals to 0.0406 s^−1^, which increased 0.0003 s^−1^. According to Equation (15), $\Delta (\frac{1}{t})$ equals to 0.0018 s^−1^, and the theoretical result $\Delta (\frac{1}{t}{)}^{\prime }$ equals to 0.0023 s^−1^, which increased 0.0005 s^−1^. Comparing Variation 1 with Variation 2, the variation trends of *f*, Δf, $\frac{1}{t}$, and $\Delta (\frac{1}{t})$ measured in experiments are all consistent with calculations.

**Figure 11:**
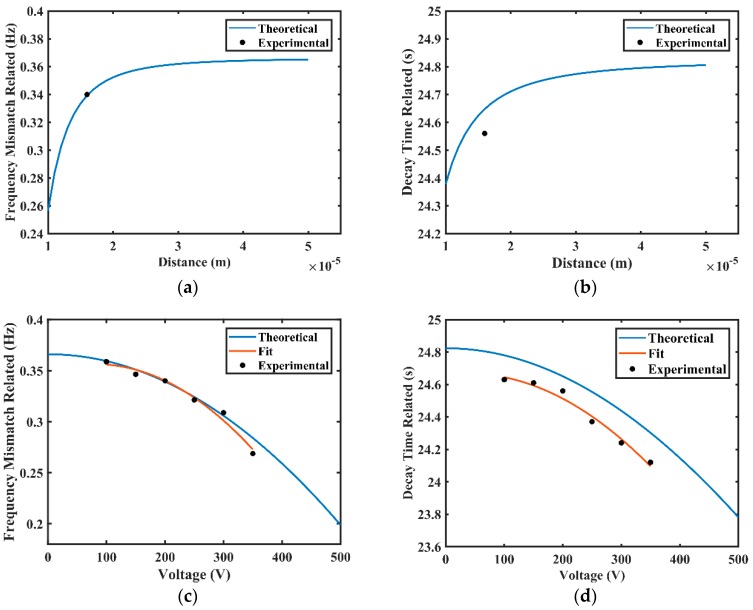
The theoretical estimations of frequency mismatch related $\Delta f^{\prime }$ and the decay time related $t^{\prime }$ of the resonator varying with the applied voltage or the distance between two electrodes: (**a**) The frequency mismatch related $\Delta f^{\prime }$ varies with the applied voltage; (**b**) The decay time-related $t^{\prime }$ varies with the applied voltage; (**c**) The frequency mismatch related $\Delta f^{\prime }$ varies with the distance between two electrodes; (**d**) The decay time-related $t^{\prime }$ varies with the distance between two electrodes.

According to the theoretical analysis, $\Delta f^{\prime }$ and $t^{\prime }$ vary with the distance between two electrodes, as shown in Figure 11a,b. As the distance between two electrodes varying from 1 × 10^−5^ m to 5 × 10^−5^ m, the frequency mismatch related $\Delta f^{\prime }$ gradually rises from 0.260 Hz to 0.362 Hz (increased by 39.23%), and the decay time related $t^{\prime }$ gradually rises from 24.38 s to 24.80 s (increased by 1.72%). Therefore, higher distance results in higher frequency mismatch and higher decay time. As the gyroscope performance degrades, when the frequency mismatch increases and decay time decreases [10], the distance between two electrodes should be set to an appropriate value for a given structure. In addition, $\Delta f^{\prime }$ and $t^{\prime }$ also vary with the applied voltage, as shown in Figure 11c,d. When the applied voltage varies from 0 V to 500 V, the frequency mismatch $\Delta f^{\prime }$ gradually decreases from 0.366 Hz to 0.205 Hz (decreased by 43.99%), and $t^{\prime }$ gradually decreases from 24.82 s to 23.78 s (decreased by 4.19%). Therefore, higher applied voltage results in lower frequency mismatch and lower decay time, the applied voltage should be optimized for a given structure. The frequency mismatch related Δf and the decay time-related *t* in practice were tested under different applied voltages, and the results are shown in Table 6. Experimental results are also displayed in Figure 11, which show good agreement with theoretical calculations.

## 5. Conclusions

This paper reports the experimental results on the changes of resonant frequency, frequency mismatch, decay time, and decay time split under electrostatic forces. Experiments were performed on a film-coated fused quartz cylindrical resonator with ring electrodes. Compared with results measured by Laser Doppler vibrometer, these parameters changed slightly with electrostatic excitation and detection. With the influence of electrostatic forces, the resonant frequency decreased by 0.022 Hz, the frequency mismatch decreased by 0.026 Hz, the decay time decreased by 0.205 s, and the decay time split increased by 0.111 s. These changes were theoretically analyzed by introducing electrostatic force into dynamic equations of the Coriolis vibratory gyroscope, and variation trends in experimental results were consistent with the theoretical analysis. The change of the frequency split and decay time with the capacitive gap and the applied voltage were estimated, and the change of the frequency split and decay time were tested under different applied voltages. Lower capacitive gaps and larger applied voltages result in lower frequency mismatch and lower decay time. Therefore, the capacitive gap and applied voltage should be appropriately designed to improve gyroscope performance.

## Figures and Tables

**Table 1:** Parameters of the resonator and the electrostatic excitation and detection system.

Component	Value	Units
*a*	1.3 × 10^−2^	m
*d*	1.6 × 10^−5^	m
*L*	5.7	mm
*l*	3.1	mm
*h*	1.2	mm
*m*	2.8 × 10^−3^	kg
*E*	200	V
*ζ_φ_*	3/13	rad
*ζ_Θ_*	π/9	rad
*φ_k_*	π/2–3/26	rad
*R*	8	GΩ

**Table 2:** The VCMs detected by the laser Doppler vibrometer.

Component	Frequency (Hz)	Decay Time (s)	Q Factor
1	7473.767	25.385	5.960 × 10^5^
2	7474.133	24.286	5.703 × 10^5^
Δ	0.366	1.099	2.578 × 10^4^

**Table 3:** The VCPs detected by electrostatic excitation and detection.

Component	Frequency (Hz)	Decay Time (s)	Q Factor
1	7473.745	25.180	5.912 × 10^5^
2	7474.085	23.970	5.628 × 10^5^
Δ	0.340	1.210	2.838 × 10^4^

**Table 4:** Results and comparisons between the VCMs and the VCPs.

Component	VCMs	VCPs	Variation
*f*_1_ (Hz)	7473.767	7473.745	−0.022
*f*_2_ ‒ *f*_1_ (Hz)	0.366	0.340	−0.026
*t*_1_ (s)	25.385	25.180	−0.205
*t*_2_ ‒ *t*_1_ (s)	1.099	1.210	0.111

**Table 5:** Results and comparisons among the theoretical vibrational characteristics, the VCPs, and the VCMs.

Component	VCMs	VCPs	Variation 1	Theoretical	Variation 2
*f* (Hz)	7473.950	7473.915	−0.035	7473.927	−0.023
Δf(Hz)	0.366	0.340	−0.026	0.339	−0.027
$\frac{1}{t}$(s^−1^)	0.0403	0.0407	0.0004	0.0406	0.0003
$\Delta (\frac{1}{t})$(s^−1^)	0.0018	0.0020	0.0002	0.0023	0.0005

**Table 6:** The frequency mismatch related Δf and the decay time-related *t* in practice varying with the applied voltage.

Voltage (V)	Δf(Hz)	*t* (s)
100	0.359	24.630
150	0.346	24.610
200	0.340	24.560
250	0.321	24.370
300	0.309	24.240
350	0.279	24.120

## Appendix A

According to Equation (4), the kinetic energy term in the Lagrangian is approximated to

(A1) $$T=\frac{1}{2}\rho ha^{2}\int_{0}^{\pi /2}\sin\varphi d\varphi \int_{0}^{2\pi }\left({\dot{w}}^{2}+{\dot{u}}_{\varphi }{}^{2}+{\dot{u}}_{\Theta }{}^{2}\right)d\Theta$$

where ρ is the density of shell material and *h* is the shell thickness. Putting in the (1), (2), (3), and the Rayleigh approximation according to Rayleigh’s Theory of Sound [49], we get

(A2) $$T=\frac{1}{2}f(2)\frac{\pi }{4}\rho ha^{2}({\dot{w}}_{c}{}^{2}+{\dot{w}}_{s}{}^{2})$$

where $f(2)=\int_{0}^{\pi /2}\sin\varphi d\varphi \left[{\left(2+\cos\varphi \right)}^{2}+2\sin^{2}\varphi \right]\tan^{4}\frac{\varphi }{2}=1.5296$, compared with (4), so $m_{eff}=\frac{f(2)}{8}m$.

The generalized oscillator equations of CVGs are

(A3) $$\begin{array}{l} \ddot{x}-k(2Ω\dot{y}+\dot{Ω}y)+\frac{2}{t}\dot{x}+\Delta (\frac{1}{t})(\dot{x}\cos2\theta_{\tau }+\dot{y}\sin2\theta_{\tau })\\ +\omega^{2}x-\omega \Delta \omega (x\cos2\theta_{\omega }+y\sin2\theta_{\omega })=f_{x}\\ \ddot{y}+k(2Ω\dot{x}+\dot{Ω}x)+\frac{2}{t}\dot{y}-\Delta (\frac{1}{t})(-\dot{x}\cos2\theta_{\tau }+\dot{y}\sin2\theta_{\tau })\\ +\omega^{2}y-\omega \Delta \omega (-x\cos2\theta_{\omega }+y\sin2\theta_{\omega })=f_{y} \end{array}$$

where $\theta_{\omega }$ is the azimuth of normal mode axis and $\theta_{\tau }$ is the azimuth of damping axis.

## Appendix B

Considering the gap between the two plates is *d* and the middle surface radius is *a*. Using the parallel plate approximation [50], the capacitance between the electrode and the resonator is

(A4) $$C=\varepsilon_{0}a^{2}\iint \frac{\sin\varphi d\varphi d\Theta }{d-w(\varphi ,\Theta ,t)}=\frac{\varepsilon_{0}a^{2}}{d}\iint \sin\varphi d\varphi d\Theta [1+\frac{w}{d}+\frac{w^{2}}{d^{2}}+\cdots ]$$

where $w(\varphi ,\Theta ,t)=(1+\frac{1}{2}\cos\varphi )tan^{2}\frac{\varphi }{2}(w_{c}(t)cos2\Theta +w_{s}(t)sin2\Theta )$, and $\varepsilon_{0}$ is the permittivity of vacuum. Expanding Equation (A4) with *w*_c_/*d* and *w_s_*/*d*,

(A5) $$\begin{array}{l} \frac{C_{k}}{C_{0}}=1+\frac{\gamma_{1}}{d}\frac{\sin\zeta_{\Theta }}{\zeta_{\Theta }}(w_{c}\cos2\Theta_{k}+w_{s}\sin2\Theta_{k})\\ +\frac{1}{2}\frac{\gamma_{2}}{d^{2}}\left\{w_{c}{}^{2}+w_{s}{}^{2}+\frac{\sin2\zeta_{\Theta }}{2\zeta_{\Theta }}\left[(w_{c}{}^{2}-w_{s}{}^{2})cos4\Theta_{k}+2w_{c}w_{s}\sin4\Theta_{k}\right]\right\} \end{array}$$

where $C_{0}=\frac{\varepsilon_{0}a^{2}\zeta_{\varphi }\zeta_{\Theta }}{d}\frac{\sin\frac{\zeta_{\varphi }}{2}}{\frac{\zeta_{\varphi }}{2}}\sin\varphi_{k}$, $\gamma_{1}=\frac{1}{2\sin\varphi_{k}\sin\frac{\zeta_{\varphi }}{2}}\mathrm{In}(\frac{1+\cos(\varphi_{k}-\frac{\zeta_{\varphi }}{2})}{1+\cos(\varphi_{k}+\frac{\zeta_{\varphi }}{2})})-\frac{1}{2}\cos\varphi_{k}\cos\frac{\zeta_{\varphi }}{2}$, $\begin{array}{l} \gamma_{2}=\frac{1}{12}\left[\cos^{2}(\varphi_{k}-\frac{\zeta_{\varphi }}{2})+\cos(\varphi_{k}-\frac{\zeta_{\varphi }}{2})\cos(\varphi_{k}+\frac{\zeta_{\varphi }}{2})+\cos^{2}(\varphi_{k}+\frac{\zeta_{\varphi }}{2})\right]-1\\ +\frac{1}{\cos(\varphi_{k}-\frac{\zeta_{\varphi }}{2})-\cos(\varphi_{k}+\frac{\zeta_{\varphi }}{2})}\mathrm{In}(\frac{1+\cos(\varphi_{k}-\frac{\zeta_{\varphi }}{2})}{1+\cos(\varphi_{k}+\frac{\zeta_{\varphi }}{2})})\\ +\frac{1}{\left[1+\cos(\varphi_{k}-\frac{\zeta_{\varphi }}{2})\right]\left[1+\cos(\varphi_{k}+\frac{\zeta_{\varphi }}{2})\right]} \end{array}$.

According to Equation (5), we seek a solution to the circuit equation of the form, so

(A6) $$q_{k}=q_{k0}+\frac{1}{R}q_{k1}+\frac{1}{R^{2}}q_{k2}+\cdots$$

Putting (A6) into (5), we get ${\dot{q}}_{k0}=0$, ${\dot{q}}_{k1}=E-\frac{q_{k0}}{C_{k}}$, ${\dot{q}}_{k2}=-\frac{q_{k1}}{C_{k}}$. Therefore, we get

(A7) $${\dot{q}}_{k1}=E-\frac{q_{k0}}{C_{0}}\left[1+(\frac{C_{0}}{C_{k}}-1)\right]$$

where $q_{k0}$ = constant, if requiring no secular term, $q_{k0}=C_{0}E$.

According to (A5), it satisfies the linear approximation

(A8) $$\frac{C_{0}}{C_{k}}=1-\frac{\gamma_{1}}{d}\frac{\sin\zeta_{\Theta }}{\zeta_{\Theta }}(w_{c}\cos2\Theta_{k}+w_{s}\sin2\Theta_{k})$$

Putting (A8) into (A7), and integrating (A7), we get

(A9) $$q_{k1}=\frac{\gamma_{1}E}{d}\frac{\sin\zeta_{\Theta }}{\zeta_{\Theta }}(cos2\Theta_{k}\int w_{c}dt+\sin2\Theta_{k}\int w_{s}dt)$$

Since $w_{c}\approx -{\ddot{w}}_{c}/\omega^{2}$, so $\int w_{c}dt\approx -{\dot{w}}_{c}/\omega^{2}$, and therefore we have

(A10) $$q_{k1}=-\frac{\gamma_{1}E}{\omega^{2}d}\frac{\sin\zeta_{\Theta }}{\zeta_{\Theta }}({\dot{w}}_{c}cos2\Theta_{k}+{\dot{w}}_{s}\sin2\Theta_{k})$$

So, the solution for $q_{k}$ can be written

(A11) $$q_{k}=C_{0}E\left[1-\frac{1}{RC_{0}\omega^{2}d}\frac{\gamma_{1}\sin\zeta_{\Theta }}{\zeta_{\Theta }}({\dot{w}}_{c}\cos2\Theta_{k}+{\dot{w}}_{s}\sin2\Theta_{k})\right]$$

According to (A5), we get

(A12) $$\begin{array}{l} \left(\frac{C_{0}}{C_{k}}-1)=-\frac{\gamma_{1}}{d}\frac{\sin\zeta_{\Theta }}{\zeta_{\Theta }}(w_{c}\cos2\Theta_{k}+w_{s}\sin2\Theta_{k}\right)\\ -\frac{1}{2}\frac{1}{d^{2}}(\gamma_{2}-\gamma_{1}{}^{2}\frac{\sin^{2}\zeta_{\Theta }}{\zeta_{\Theta }{}^{2}})(w_{c}{}^{2}+w_{s}{}^{2})\\ -\frac{1}{2}\frac{1}{d^{2}}(\gamma_{2}\frac{\sin2\zeta_{\Theta }}{2\zeta_{\Theta }}-\gamma_{1}{}^{2}\frac{\sin^{2}\zeta_{\Theta }}{\zeta_{\Theta }{}^{2}})\left[(w_{c}{}^{2}-w_{s}{}^{2})\cos4\Theta_{k}+2w_{c}w_{s}\sin4\Theta_{k}\right] \end{array}$$

Therefore, from Equation (A12), we get

(A13) $$\begin{array}{l} \frac{\partial }{\partial w_{c}}(\frac{C_{0}}{C_{k}})=-\frac{\gamma_{1}}{d}\frac{\sin\zeta_{\Theta }}{\zeta_{\Theta }}\cos2\Theta_{k}-\frac{1}{d^{2}}(\gamma_{2}-\gamma_{1}{}^{2}\frac{\sin^{2}\zeta_{\Theta }}{\zeta_{\Theta }{}^{2}})w_{c}\\ -\frac{1}{d^{2}}(\gamma_{2}\frac{\sin2\zeta_{\Theta }}{2\zeta_{\Theta }}-\gamma_{1}{}^{2}\frac{\sin^{2}\zeta_{\Theta }}{\zeta_{\Theta }{}^{2}})(w_{c}\cos4\Theta_{k}+w_{s}\sin4\Theta_{k}) \end{array}$$

(A14) $$\begin{array}{l} \frac{\partial }{\partial w_{s}}(\frac{C_{0}}{C_{k}})=-\frac{\gamma_{1}}{d}\frac{\sin\zeta_{\Theta }}{\zeta_{\Theta }}\sin2\Theta_{k}-\frac{1}{d^{2}}(\gamma_{2}-\gamma_{1}{}^{2}\frac{\sin^{2}\zeta_{\Theta }}{\zeta_{\Theta }{}^{2}})w_{s}\\ +\frac{1}{d^{2}}(\gamma_{2}\frac{\sin2\zeta_{\Theta }}{2\zeta_{\Theta }}-\gamma_{1}{}^{2}\frac{\sin^{2}\zeta_{\Theta }}{\zeta_{\Theta }{}^{2}})(-w_{c}\sin4\Theta_{k}+w_{s}\cos4\Theta_{k}) \end{array}$$

Putting (A11), (A13), (A14) together, we get

(A15) $$\begin{array}{l} \frac{q_{k}{}^{2}}{2C_{0}}\frac{\partial }{\partial w_{c}}(\frac{C_{0}}{C_{k}})=-\frac{C_{0}E^{2}}{2d^{2}}\\ \left[\left(\gamma_{2}-\gamma_{1}{}^{2}\frac{\sin^{2}\zeta_{\Theta }}{\zeta_{\Theta }{}^{2}})w_{c}+(\gamma_{2}\frac{\sin2\zeta_{\Theta }}{2\zeta_{\Theta }}-\gamma_{1}{}^{2}\frac{\sin^{2}\zeta_{\Theta }}{\zeta_{\Theta }{}^{2}})(w_{c}\cos4\Theta_{k}+w_{s}\sin4\Theta_{k}\right)\right]\\ +\frac{\gamma_{1}{}^{2}E^{2}}{2R\omega^{2}d^{2}}\frac{\sin^{2}\zeta_{\Theta }}{\zeta_{\Theta }{}^{2}}\left[{\dot{w}}_{c}+{\dot{w}}_{c}\cos4\Theta_{k}+{\dot{w}}_{s}\sin4\Theta_{k}\right] \end{array}$$

(A16) $$\begin{array}{l} \frac{q_{k}{}^{2}}{2C_{0}}\frac{\partial }{\partial w_{s}}(\frac{C_{0}}{C_{k}})=-\frac{C_{0}E^{2}}{2d^{2}}\\ \left[\left(\gamma_{2}-\gamma_{1}{}^{2}\frac{\sin^{2}\zeta_{\Theta }}{\zeta_{\Theta }{}^{2}})w_{s}-(\gamma_{2}\frac{\sin2\zeta_{\Theta }}{2\zeta_{\Theta }}-\gamma_{1}{}^{2}\frac{\sin^{2}\zeta_{\Theta }}{\zeta_{\Theta }{}^{2}})(-w_{c}\sin4\Theta_{k}+w_{s}\cos4\Theta_{k}\right)\right]\\ +\frac{\gamma_{1}{}^{2}E^{2}}{2R\omega^{2}d^{2}}\frac{\sin^{2}\zeta_{\Theta }}{\zeta_{\Theta }{}^{2}}\left[{\dot{w}}_{s}-(-{\dot{w}}_{c}\sin4\Theta_{k}+{\dot{w}}_{s}\cos4\Theta_{k})\right] \end{array}$$

And the complete expression of *A* is $m_{eff}[\Delta (\frac{1}{t})(\cos2\theta_{\tau }{\dot{w}}_{c}+\sin2\theta_{\tau }{\dot{w}}_{s})-\omega \Delta \omega (\cos2\theta_{\omega }w_{c}+\sin2\theta_{\omega }w_{s})]$.
